# Circulating cancer stem cells: an interesting niche to explore

**DOI:** 10.37349/etat.2020.00016

**Published:** 2020-08-31

**Authors:** Federica Papaccio

**Affiliations:** Department of Medicine, Surgery and Dentistry “Scuola Medica Salernitana”, University of Salerno, Via S. Allende, 84081 Baronissi (SA), Italy; Università degli studi della Campania, Italy

Cancer stem cells (CSCs) constitute a relevant subpopulation of cells within the tumor from the beginning of its development. They are the tumor initiating cells, capable of self-renewal and multiple differentiating potential [1–3]; these particular cells are responsible for resistance to radiotherapy and chemotherapy, and ultimately disease recurrence and progression in cancer patients [4]. As a consequence, eradicating this subpopulation would be critical in order to achieve patient’s cure [5, 6].

Circulating tumor cells (CTCs) are cancer cells that are shed into the blood system even at very early stage of cancer development [7], being a promising tool for detection of minimal residual disease and progression [8–10]. Their concentration correlates with high tumor burden, offering a prognostic tool [11]. Nevertheless, CTCs detection and isolation still remains challenging from a technical point of view, particularly when searching for a standardized reproducible method, which is critical when looking for a clinical application [12–14]. Indeed, despite all the efforts, CTCs exploration has not been widely diffused. The only FDA approved technology for CTCs detection is CellSearch, since 1999 [15]. This is an immune-affinity enrichment method, based on the detection of the epithelial cell adhesion membrane (EpCAM) protein.

Some experiences are published about the detection of CTCs in lung cancer patients as a diagnostic and prognostic tool [16–19]. Actually, liquid biopsy is an interesting field of investigation in lung cancer for several important reasons. Lung cancer is the leading cause of cancer related deaths worldwide, it is often diagnosed in advanced stage; moreover, despite the importance of molecular profiling of these tumors in order to offer a personalized treatment, often tissue biopsies are technically difficult. Indeed, CTCs isolation and characterization for driver mutations is feasible and could offer a clear advantage over classical tissue biopsies [20], increasing the number of patients who can access to molecularly-guided therapy.

Interestingly, some experimental findings have highlighted that a small proportion of CTCs displays CSCs features (tumor initiating capability, in particular), so that they can be considered as circulating tumor stem cells (CTSCs) [21–23]. For this reason, the investigation towards the identification of CTCs and their potential role in metastasis has increased over the last years [24–26]. This finding holds important implications as stem population is highly tumorigenic and so targeting it could be crucial towards the prevention of disease progression [27]. Indeed, to improve CTSCs isolation is of outmost importance.

CTSCs have been identified in several solid tumors, such as colorectal cancer [28] and breast cancer [19].

A preliminary study combined different surface markers including epithelial (cytokeratin and EpCAM) and CSC markers [CD44, Aldehyde dehydrogenase (ALDH), and CD24] aiming at increase the sensitivity of the assay over a one marker approach [29]. This study has been conducted on CTCs isolated from peripheral blood of non-small cell lung cancer (NSCLC) patients and has been functionally validated: the CTSCs were able to form pleurospheres *in vitro*, which is among the definition criteria of CSCs [30].

The potential prognostic value was already highlighted in a 2011 paper where CTSCs predicted recurrence in hepatocellular carcinoma patients after surgical resection [31]. In a later paper, circulating CD44+ CSCs could predict risk of recurrence in gastric cancer patients [32].

Actually, CD44+ CTSCs have been identified in NSCLC patients. Their levels were inversely correlated with serum tumor necrosis factor-related apoptosis-inducing ligand (sTRAIL) levels, which have been associated with a tumor suppressive effect [33]. In another study, sTRAIL was negatively correlated with ALDH1+ cells, the latter being a different marker which can be used for NSCLC CTSCs characterization [34].

Another point to be considered is that within CTSCs there are distinct sub-populations, and that those expressing epithelial-mesenchymal transition (EMT) features could be an even more aggressive subtype [35]. The coexistence of CSC and EMT markers has been demonstrated on breast cancer CTCs [19, 36, 37]. Indeed, this complex phenotype prevails in more advanced and metastatic cases [37]. In addition, it was demonstrated that this subpopulation is more drug resistant than the other subtypes [38].

The heterogeneity of cell populations can be detected also among CTSCs that display mesenchymal or epithelial phenotypes: in a cohort of 43 NSCLC patients it was detected the presence of both epithelial and mesenchymal CTSCs subpopulations. Cells with mesenchymal features had a phenotype that correlated with stemness and with a more aggressive behavior, including a reduced progression free survival [39]. On the other hand, the phenotypic heterogeneity of CTSCs could encourage the use of multiple markers in order to allow a better caption of all the stem population [40].

New insights on CSTCs will surely come from single-cell technology studies. A recently published study employed a multigene nanoplatform to evaluate gene expression on hundreds of single CTCs from lung cancer patients [41].

CTCs from lung cancer patients bear an incredible potential, from gene mutation to gene expression, and could help to capture cancer heterogeneity and evolution when analyzed as single cell. Thus, they could be of potential relevance for progression monitoring and molecularly guided treatment decision, improving precision medicine. Tremendous applications are represented by the possibility to isolate CTCs for cell culture, which allowed to create CTC-derived xenografts [42] in animal models and more recently organoids culture [43], for drug screening as an example. Clearly, this constitutes an added value compared with other liquid biopsy techniques, relying for example on circulating DNA isolation. Nevertheless, there are several issues to consider: first of all, the fact that CTCs and even more CSTCs are difficult and rare to detect [44]. The majority of enrichment methods are based on the epithelial marker EpCAM, which is often not expressed in the stem/EMT compartment. Furthermore, for cell culture it is needed a system capable of ensuring a high viability of cells after sorting [45]. Emerging enrichment technologies are employing electrochemical methods, thus avoiding the selection of surface markers [46, 47].

Certainly, more attention should be focused on the possibility to isolate the stem/EMT population directly from the patient, as it has been showed in a significant breast cancer patients’ cohort [48] and possibly in the future to perform functional studies on this specific cells. This approach could allow for instance to screen drugs on putative stem-related oncogenic vulnerabilities [49, 50].

## Abbreviations

ALDH: Aldehyde dehydrogenase
CSCs: cancer stem cells
CTCs: circulating tumor cells
CTSCs: circulating tumor stem cells
EMT: epithelial-mesenchymal transition
EpCAM: epithelial cell adhesion membrane
NSCLC: non-small cell lung cancer
sTRAIL: serum tumor necrosis factor-related apoptosis-inducing ligand

## Declarations

### Copyright

© The Author(s) 2020.
